# Zika virus infection modulates the metabolomic profile of microglial cells

**DOI:** 10.1371/journal.pone.0206093

**Published:** 2018-10-25

**Authors:** Fodé Diop, Thomas Vial, Pauline Ferraris, Sineewanlaya Wichit, Michèle Bengue, Rodolphe Hamel, Loïc Talignani, Florian Liegeois, Julien Pompon, Hans Yssel, Guillaume Marti, Dorothée Missé

**Affiliations:** 1 MIVEGEC UMR 224, Univ Montpellier, IRD, CNRS, Montpellier, France; 2 PHARMADEV UMR 152, Université de Toulouse, IRD, Toulouse, France; 3 Department of Clinical Microbiology and Applied Technology, Faculty of Medical Technology, Mahidol University, Salaya, Thailand; 4 Centre d’Immunologie et des Maladies Infectieuses, Inserm, U1135, Sorbonne Universités, UPMC, APHP Hôpital Pitié-Salpêtrière, Paris, France; Loyola University Chicago, UNITED STATES

## Abstract

Zika virus (ZIKV) is an emerging arbovirus of the Flaviviridae family. Although infection with ZIKV generally leads to mild disease, its recent emergence in the Americas has been associated with an increase in the development of the Guillain-Barré syndrome in adults, as well as with neurological complications, in particular congenital microcephaly, in new-borns. To date, little information is available on neuroinflammation induced by ZIKV, notably in microglial cells in the context of their metabolic activity, a series of chemical transformations that are essential for their growth, reproduction, structural maintenance and environmental responses. Therefore, in the present study we investigated the metabolomic profile of ZIKV-infected microglia. Microglial cells were exposed to ZIKV at different time points and were analyzed by a Liquid Chromatography-High Resolution mass spectrometry-based metabolomic approach. The results show that ZIKV infection in microglia leads to modulation of the expression of numerous metabolites, including lysophospholipids, particulary Lysophosphatidylcholine, and phospholipids such as Phosphatidylcholine, Phosphatidylserine, Ceramide and Sphingomyelin, and carboxylicic acids as Undecanedioic and Dodecanedioic acid. Some of these metabolites are involved in neuronal differentiation, regulation of apoptosis, virion architecture and viral replication. ZIKV infection was associated with concomitant secretion of inflammatory mediators linked with central nervous system inflammation such as IL-6, TNF-α, IL-1β, iNOS and NO. It also resulted in the upregulation of the expression of the gene encoding CX3CR1, a chemokine receptor known to regulate functional synapse plasticity and signaling between microglial cells. These findings highlight an important role for microglia and their metabolites in the process of neuroinflammation that occurs during ZIKV pathogenesis.

## Introduction

Zika virus (ZIKV) is a newly emerging arbovirus of the *Flaviviridae* family that is related to other medically important flaviviruses, such as Dengue, Yellow Fever, and West Nile[1]. ZIKV was responsible for two main outbreaks in Yap Island and French Polynesia in 2007 and 2014, respectively, and subsequently has spread to South and Central America where it caused a widespread epidemics [2]. The expansion of ZIKV on the American continent emphasizes the capacity of ZIKV to spread to non-endemic regions worldwide. A phylogenetic analysis of the virus circulating in Latin America shows that it belongs to the same Asian lineage that circulated in French Polynesia [3]. Whereas adults infected by ZIKV usually suffer only from mild clinical symptoms, numerous cases of neurological disorders and congenital manifestations were reported after the outbreak in the Americas, which transformed the Zika threat into a worldwide public health emergency [4]. In particular, an unusual increase in Guillain-Barré syndrome concomitant to ZIKV circulation was reported in French Polynesia and several countries in Latin America [5], as well as a sharp rise in the incidence of pregnancy-associated microcephaly linked to ZIKV infection that occurred between 2014 and 2016 [6]. There is strong evidence indicating that ZIKV infection in pregnant women causes congenital abnormalities and fetal demise [7]. Viral RNA and antigen in the brains of infected fetuses and newborns have been detected in cases of microcephaly [8]. Moreover, ZIKV often causes spontaneous abortions in infected mothers. One potential mechanism for the observed microcephaly is the capacity of ZIKV to preferentially infect human neural progenitor cells and to trigger apoptosis in these cells [9]. In addition, infection of human neurosphere organoïd cultures *in vitro* with ZIKV reportedly impairs their growth and increases cell death [10][11]. Results from a recent study showed that microglia interact with ZIKV-infected human tissues and contribute to further spreading of the virus [12] which corroborates a report showing that microglia are one of the main targets of ZIKV in the developing brain [13]. This notion is underscored by our recent observation that ZIKV infects human microglial cells and causes the emergence of supernumerary foci with centriolar proteins and impaired spindle positioning [14].

Microglia are mononuclear phagocytes that play an important role in neuronal development, as well as in the homeostasis of the central nervous system, and that have a marked impact on normal brain functioning and maintenance of tissue integrity [15]. An important molecule in the homeostatic function of microglia is CX3CR1, since interaction of this chemokine receptor with its unique ligand, CX3CL1 has been reported to regulate axon outgrowth during embryogenesis. In addition, CX3CR1 signaling controls microglial density within neural circuits, which, in turn, modulates synaptic pruning and maturation [16]. Microglia are also an important source of inflammatory factors the production of which is associated with various neuronal pathologies [17]. Activation of microglia leads to the production of pro-inflammatory cytokines like tumor necrotic factor-α (TNF-α), interleukin-1β (IL-1β), IL-6, IL-12, and cytotoxic molecules such as nitric oxide (NO) that aggravate the inflammatory damage [18].

In the central nervous system (CNS), increased levels of metabolites such as lysophospholipids have been found under various pathological conditions and their expression has been linked to neurodegeneration [19]. Lysophosphatiylcholine (LPC) is involved in neuroinflammation by activating the inflammasome pathway in microglia [20]. In addition, LPC induces pericyte loss and apoptosis [21]. Phospholipids are of particular importance for the CNS. It has been shown that the synthesis of adequate amount of PC is necessary for neuronal differentiation and alterations of sphyngolipids levels have been associated with neurodegenerative diseases [22][23]. However, the role of metabolites in neurological complications as a result of ZIKV infection has not been addressed as yet.

Therefore, there is a need to investigate the metabolomics profile of ZIKV-infected microglial cells that could be associated with the pathogenesis of ZIKV infection. In this study, we demonstrate that ZIKV-infected microglia cells secrete several pro-inflammatory cytokines whose production is associated with that of inflammatory metabolites.

## Materials and methods

### Cells, viruses, and reagents

Vero cells (African green monkey kidney-derived cells) were grown in Dulbecco’s modified Eagle’s medium (DMEM; Invitrogen, Cergy Pontoise, France) supplemented with 5% fetal calf serum (FCS); Lonza, Basel, Switzerland). C6/36 mosquito cells, used for propagation of the ZIKV, were maintained at 28°C in DMEM supplemented with 10% FCS, as previously described [24]. The microglia CHME-5 cell line was originally obtained from human fetal microglia transfected with the large T-antigen of SV-40 [25] and grown at 37°C in DMEM supplemented with 10% FCS. The ZIKV strain PF-25013-18 was kindly provided by V. M. Cao-Lormeau (Institut Louis Malardé, French Polynesia).

### ZIKV infection of cells

For infection, cells were seeded in culture plates and grown to 70% confluence. The number cells per cm^2^ used depended on the experiment and days post infection. The cultures were rinsed with phosphate-buffered saline (PBS) and ZIKV at the desired multiplicity of infection (MOI) were added to the cells. The cells were incubated for 2 h at 37°C with gentle agitation every 30 min. Then, the inoculum was removed and the cells were washed three times with PBS. Culture medium was added to each well, and the cells were incubated at 37°C for the duration of the experiment. As a negative control, CHME-5 cells were incubated with the culture supernatant from uninfected C6/36 cells, referred to in the present study as mock-infected cells.

### Extraction of metabolites from microglial cells

Metabolites were extracted using a cold methanol/water solution at 80% as an extraction solvent which serves for the extraction of a broad spectrum of metabolites [26]. Briefly, at the appropriate time post infection, the medium was aspirated from the cultures and the cells were washed with 500 ul per well of 0.9% NaCl at room temperature, followed by the immediate addition of 80% methanol at -20°C to quench metabolic activity. The extraction of metabolites was carried out by ultrasound stimulation for 15 min on ice, then the cells residues were centrifuged at 10.000 rpm for 1min at 4°C. Subsequently the supernatant was dried using a Speedvac for approximately 5h to dry all solvent. All extracts were weighted and dissolved at 2 mg/mL in extraction solvent for further ultra-high pressure liquid chromatography mass spectrometry (UHPLC-MS) analysis.

### LC-MS based metabolomic analysis

All extracts were profiled using a UHPLC-LTQ Orbitrap XL instrument (Ultimate 3000, Thermo Fisher Scientific, Hemel Hempstead, UK) set at 15.000 resolution. Sample analysis was carried out under PI and NI mode. The mass scanning range was m/z 100–1500 and the capillary temperature was 300°C and ISpray voltage at 4.2 kV (positive mode) and 3.0 kV (negative mode). The injection volume was 3 μL. Mass measurement was externally calibrated just before begining the experiment. Each full MS scan was followed by data dependant MS/MS on the four most intense ions using stepped CID fragmentation mode at 35% normalized collision energy, isolation width of 2 ul and activation Q set at 0.250. The LC–MS system was run in binary gradient mode and each sample was injected on tow complementary analytical method to expense metabolite coverage:

Lipophilic to medium range polarity compounds were profiled using a UPLC BEH C18 Acquity column (100 × 2.1 mm i.d., 1.7 μm, Waters, MA, USA) equipped with a guard column. The mobile phase consisted of 0.1% formic acid (FA) in water (phase A) and 0.1% FA in acetonitrile (phase B). The linear gradient program was as follows: 9 A for 0.5 min to 20% B over 3.5 min, 98% B for 8 min, held at 98% B for 3 min, and returned in 0.5 min to initial conditions (98% A), finally held for 3.5 min to assure equilibration before the subsequent analysis. The flow rate was 0.4 mL/min. The column temperature was kept at 45°C. Polar metabolites were profiled using a Zic-pHilic column (150 × 2.1 mm i.d., 5 μm, SeQuant, Merck, Darmstadt, Germany). Solvent A was 20 mM ammonium acetate buffered at pH 9 and solvent B was acetonitrile; the flow rate was 0.25 ml/min. The gradient was as follows: 90% B for 0.5 min to 40% B over 18 min, held at 40% B for a further 3 min, and then returned for 0.5 min to initial conditions (90% B) finally held for 5 min for subsequent analysis. The injection volume was 3 μL.

### Data analysis

Peak detection and alignment were performed by MS-DIAL [27] (ver. 2.50). Peak identification was done using MS-finder [28] with PubChem, CheBi and HMDB databases to perform *in silico* matches. Data were normalized by total ion chromatogram (TIC) and transformed by auto-scaling. The dataset comprising all samples and normalized peak area of identified features is available as supplementary table (S1 Table). Multivariate data analysis was performed on the resulting peak matrix using SIMCA-P software (version 14.1; MKS Umetrics AB). The orthogonal projection to latent structure (OPLS) regression analysis was done according to viral titers as Y input. VIP (Variable importance for projection) scores were used to rank variables statistically linked to ZIKV infection. For each model, a leave-one-subject-out cross-validation was performed to assess the model fit. The validity of the discriminant model was verified using 100 permutation tests (Y-scrambling, S1 Fig). All raw data were deposited on MassIVE data repository and are publicly available (MassIVE ID: MSV000082945).

### Plaque assay

Four different 10-fold dilutions of viral supernatant corresponding to 6, 12, 24 and 48 hpi were spread onto monolayers of Vero cells at 37°C for 2 h to initiate binding to the cells. Then, a mix of nutriment solution with CMC (Lonza) was added. The cells were incubated at 37°C for 5 days before the plaque assay. For plaque counting, the cells were incubated with 3.7% formaldehyde and 0.1% crystal violet in 20% ethanol.

### Measurement of viral RNA levels

Extraction of RNA and measurement of viral RNA levels were carried out as described previously [29]. The primer and probe sequences targeting ZIKV have already been described [30].

### Real-time PCR analysis

cDNA was synthesized using 0.5μg of CHME-5 cells total RNA and the MMLV reverse transcription Kit, following manufacturer’s protocol (Promega, Charbonière, France). Gene expression was quantified using real-time PCR with an Applied Biosystems 7300 real-time PCR system. RT-qPCR primers were synthesized, ~100 bps based on the base sequence of GenBank (https://www.ncbi.nlm.nih.gov/genbank) and shown in Table 1. Real-time PCR was performed using 2 μl of cDNA with specific primers targeting the genes of interest and 400 nM of each primer and 4 μl of Fast Eva Green Master Mix (Invitrogen; Thermo Fisher Scientific, Inc.) in an 8 μl reaction volume. The cycling conditions were 45 cycles of 95°C for 15 s, 60°C for 15 s, and 72°C for 20 s. mRNA expression (fold change) was quantified by calculating the 2^−ΔΔ*CT*^ value, with glyceraldehyde-3-phosphate dehydrogenase (GAPDH) mRNA as an endogenous control.

**Table 1:** List of primers used in this study.

Name	Sequence (5′-3′)
**IFN-α**	ACCCACAGCCTGGATAACAG CTCTCCTCCTGCATCACACA
**IFN-β**	GAC GCC GCATTGACC ATCTA TTGGCCTTCAGGTAATGCAGAA
**IFN-γ**	TCG GTAACTGACTTGAATGTCCA TCGCTTCCCTGTTTTAGCTGC
**TNf-α**	CCT GTG AGGAGGACGAAC AT AGGCCCCAGTTTGAATTC TT
**CX3CR1**	CTCAAAGTGAGGGGAAACCA GGACGTARGAGAAGCCAAGC
**IL1-β**	AACCTCTTCGAGGCACAAGG GTCCTGGAAGGAGCACTTCAT
**IL6**	ATGAACTCCTCCTCCACAAGCGC GAAGAGCCCTCAGGCTGGACTG

### Assessment of NO production

The activity of NO was determined by measuring the nitrite (NO_2_-) concentration in the culture media using the Griess reagent system (Promega, Charbonnières-les-Bains, France). Each medium supernatant (100 ml) was mixed with 50 ml 1% sulfanilamide (in 5% phosphoric acid, Sigma-Aldrich; Merck kGaA) and 50 ml 0.1% N-(1-Naphthyl) ethylenediaminedihydrochloride (Santa Cruz Biotechnology, Inc.) and incubated in the dark at room temperature for 10 min. Absorbance was measured using a SpectraMax L Microplate Reader (Molecular Devices, LLC, Sunnyvale, CA, USA) at 540 nm. NO_2_ concentration was determined based on the nitrogen standard curve.

### Immunofluorescence analysis

Forty-eight hours following infection of CHME5 cells with ZIKV (MOI = 1), cells were washed three times and fixed with 4% paraformaldehyde in PBS for 10 min at room temperature. Cells were then permeabilized with 0.1% Triton X-100 for 10 minutes, washed in PBS, supplemented with 10% FCS, and were incubated with iNOS rabbit polyclonal antibody (Fisher Scientific, Illkirch France) at a 1:250 dilution in 10% BSA and incubated for 3 hours at room temperature. After washing three times in PBS, the cells were incubated with a donkey anti-rabbit IgG (H+L) antibody (Alexa Fluor 488 conjugate, Fisher Scientific, Illkirch, France, at a dilution of 1:2000 in 10% BSA for 45 minutes at room temperature. Cell nuclei were stained with Prolong Gold antifade mountant containing DAPI (Fisher Scientific). As a positive control, cells were treated with a combination of TNF-α (5 ng/mL) and IFN-γ (50 U/mL), both purchased from R&D Systems Europe, Lille. The images were captured at a 63 fold magnification on a Zeiss Axio Imager Z2 fluorescent microscope (Carl Zeiss, Marly-le-Roi, France).

## Results

### Microglial cells are permissive for ZIKV infection

**Fig 1:**
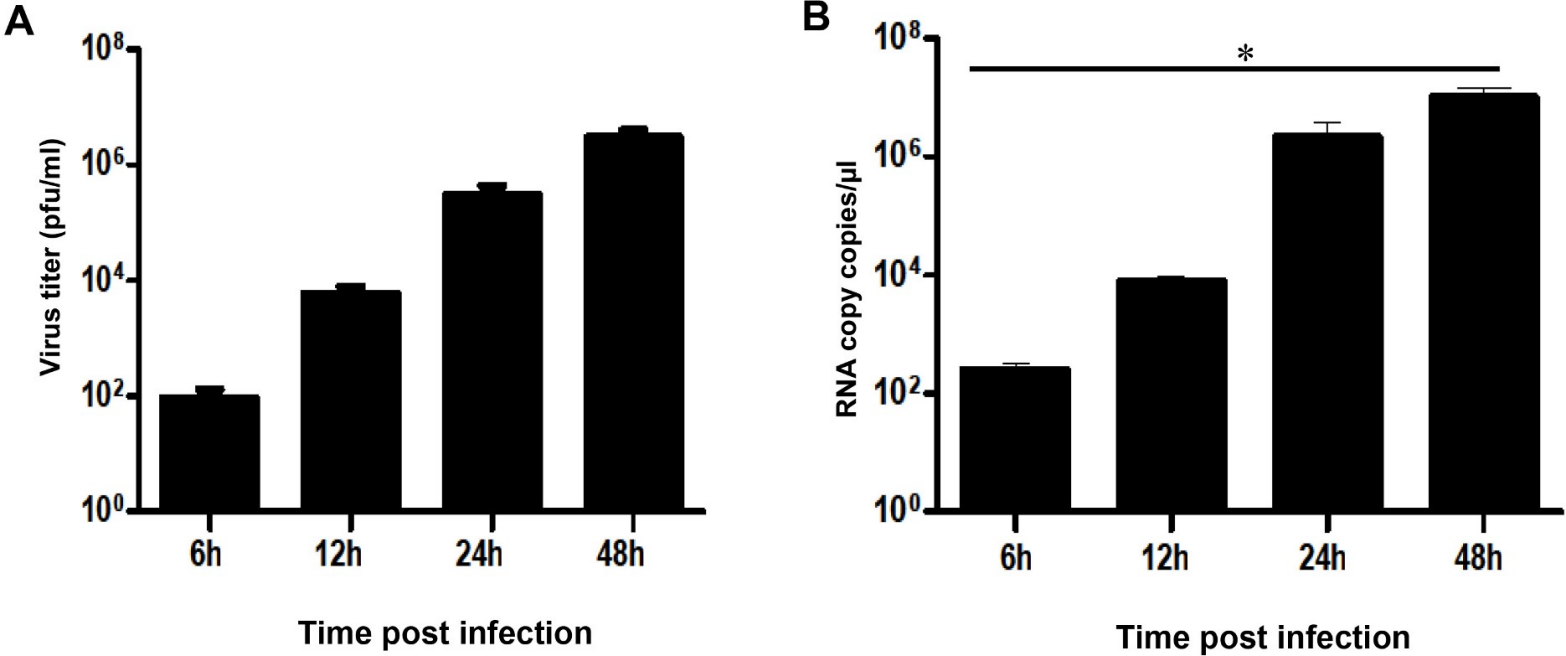
Human microglia cells are permissive to ZIKV infection. Microglia cells were infected with ZIKV (MOI = 1) and expression of viral RNA was measured at different times post-infection by real-time RT-PCR (A). Viral replication was also determined by plaque assay analysis of culture supernatants of ZIKV-infected cells (B). Experiments were performed three times and errors bars represent standard error of the mean. The *t*-test was employed to analyze the differences between sets of data. *, *P* < 0.05.

First, we tested the ability of microglia cells to produce viral progeny *in vitro* by determining viral titers in the supernatants of the ZIKV-infected cell line CHME-5, using a standard plaque assay. The results show a gradual increase in the production of viral particles in a time-dependent manner, indicating active viral replication in the infected cells (Fig 1A). Intracellular viral RNA was also detected by real-time PCR at different time points post infection (Fig 1B). Viral RNA copy numbers could be determined as soon as 6 hours post infection (hpi) and steadily increased during infection. Levels of viral transcripts were markedly high and reached up to 10^6^ RNA copies/μl at 48 hpi.

### ZIKV infection induces the production of proinflammatory cytokines and CX3CR1 expression by microglial cells

**Fig 2:**
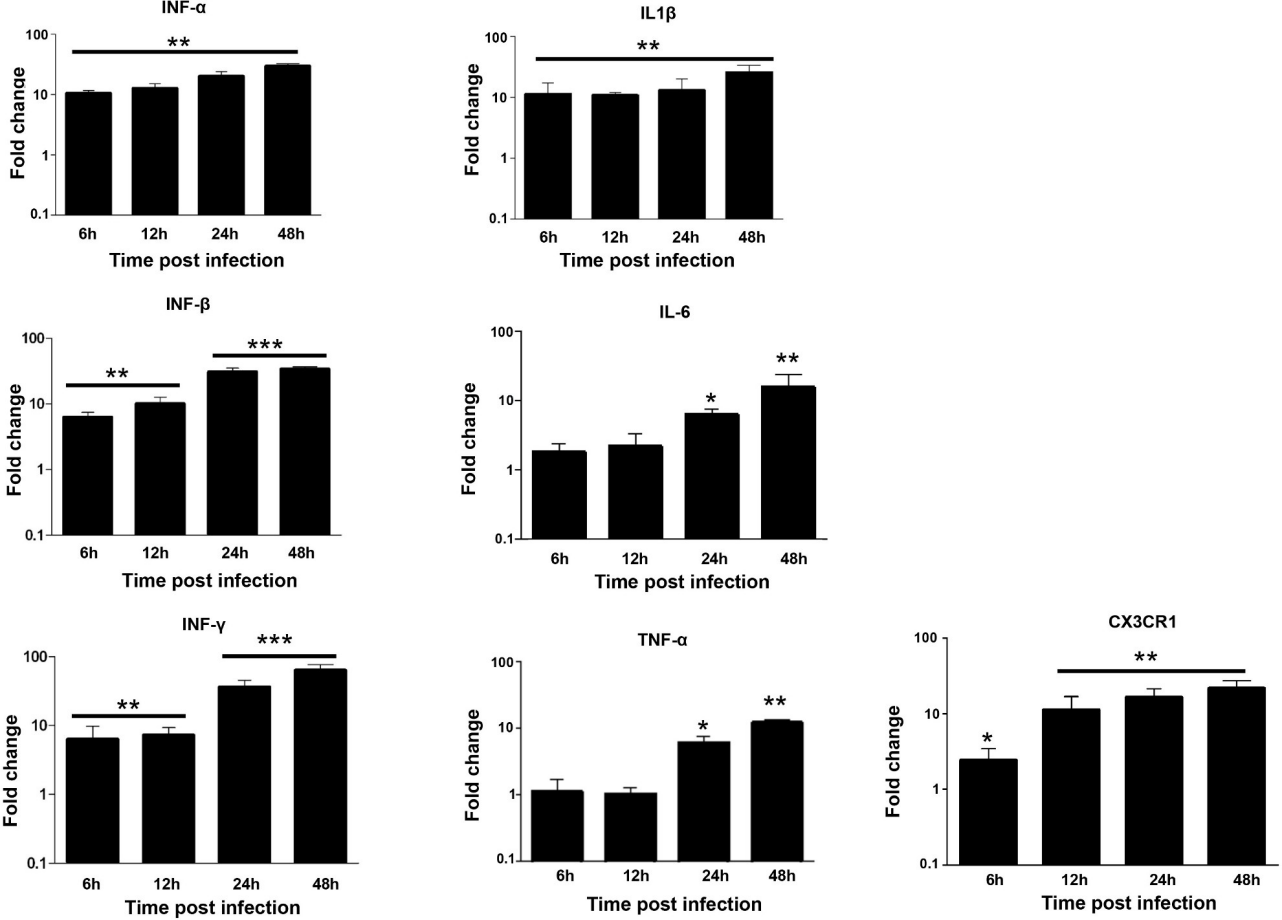
ZIKV induces the production of inflammatory mediators and CX3CR1 in microglial cells. CHME5 cells were exposed to ZIKV (MOI 1) and mRNA levels were quantified over time by real-time RT-PCR. Results are expressed as the fold induction of transcripts in ZIKV-infected cells relative to those in Mock-infected cells. Data are representative of three independent experiments, each performed in duplicate (error bars represent SEM). The Wilcoxon-Mann-Whitney test was employed to analyze the differences between sets of data. *p*-values of < 0.05 *; < 0.01**; < 0.001 *** were considered significant.

CHME-5 cells were infected with ZIKV at MOI 1 and harvested at 6, 12, 24 and 48 hpi to determine the expression of transcripts for various proinflamatory immune mediators including CX3CR1. ZIKV infection induced a clear antiviral immune response in these cells as shown by the production of high levels of IFN-α, IFN-β and IFN-γ, as well as neurotoxic factors such as TNF-α, IL-1β and IL-6 during the first hours of infection with maximum levels at 48 hpi (Fig 2). In contrast, only low expression levels of TNF-α and IL-6 were detected in the microglial cells 6 and 12 hours following infection with ZIKV (Fig 2). Moreover, the expression of CX3CR1, an important regulator of microglia function at the neuronal synapse [31] was gradually upregulated in ZIKV-infected microglial cells 6, 12, 24 and 48 hpi, as compared to mock-infected cells (Fig 2).

### ZIKV upregulates iNOS expression and nitric oxide production in microglial cells

**Fig 3:**
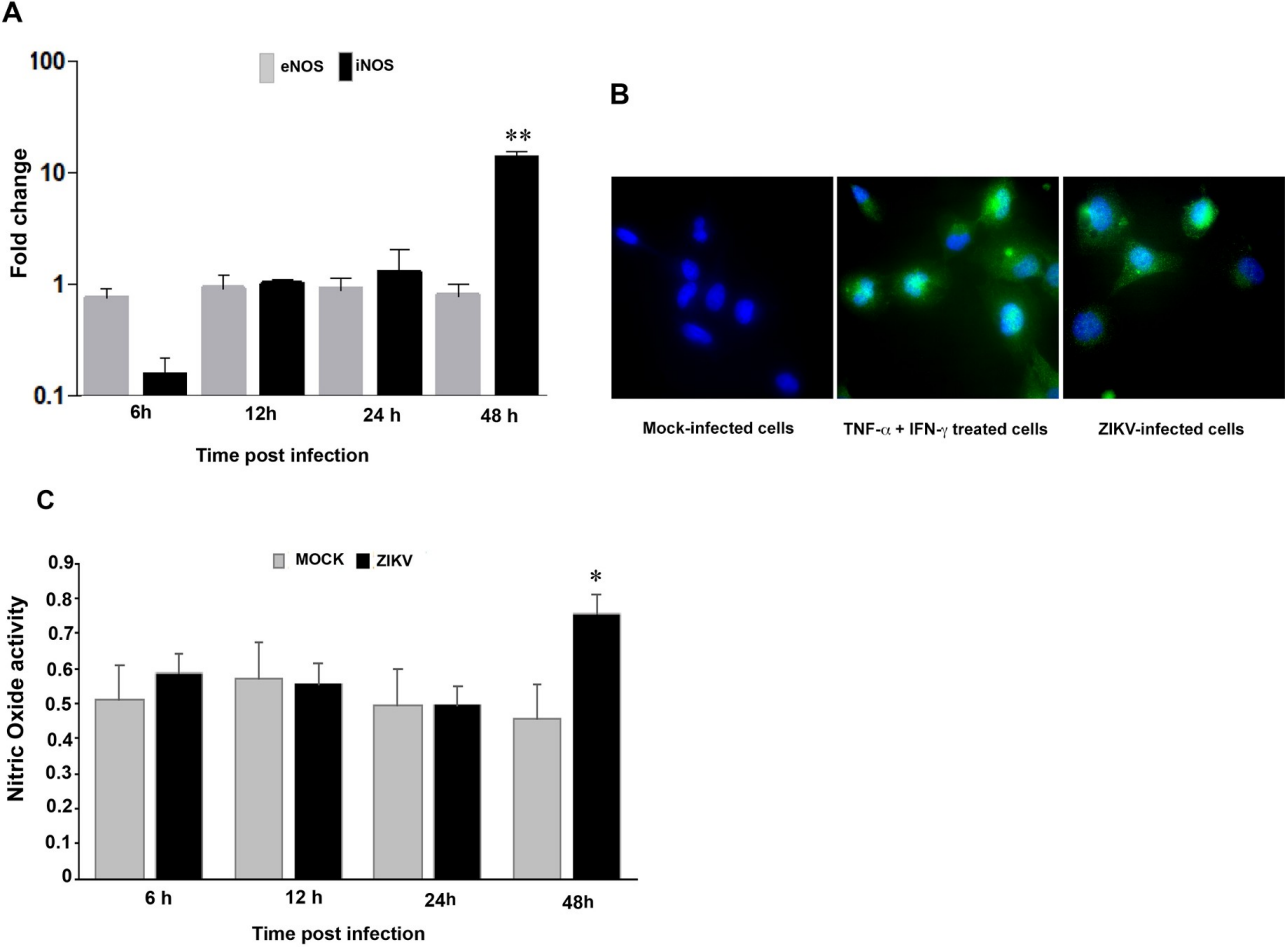
Analysis of NO in ZIKV-infected microglial cells. CHME5 cells were exposed to ZIKV (MOI 1) and expression of iNOS and eNOS was determined by real-time RT-PCR (A). Results are expressed as the fold induction of transcripts in ZIKV-infected cells, relative to those in mock-infected cells. Data are representative of four independent experiments, each performed in duplicate (error bars represent SEM). The Wilcoxon-Mann-Whitney test was used to analyze the differences between sets of data. *p*-values of < 0.05 *; < 0.01**; < 0.001 *** were considered significant. NO expression (green fluorescence) was determined by immunofluorescence in CHME5 cells infected with ZIKV at 48 hpi (B). As a positive control, cells were treated with a combination of TNF-α and IFN-γ. Staining of cell nuclei is shown as blue fluorescence. NO activity in supernatants of mock-and ZIKV-infected cells was measured using Griess reagent (C). Data are presented as the mean ± standard deviation of five independent experiments. *p*-values of <0.05 were considered significant (*).

Nitric oxide (NO) derived from microglia under the influence of proinflamatory cytokines, leads to oligodendrocyte degeneration in demyelinating diseases and neuronal death. NO is a free radical synthesized by the action of NO synthase (NOS), an enzyme existing in three isoforms. Neuronal NOS and endothelial NOS (eNOS) are responsible for the continuous basal release, whereas inducible NOS (iNOS) is regulated by inflammatory cytokines. To compare NOS expression in ZIKV infected cells, the gene expression of iNOS and eNOS were measured by real-time PCR. The eNOS mRNA expression did not change when measured at different times post infection (Fig 3A). However, iNOS mRNA levels were upregulated in ZIKV-infected microglia cells at 24 hpi, with maximal mRNA levels detected at 48 hpi (Fig 3A). The upregulation of iNOS expression in ZIKV-infected cells was confirmed by immunofluorescence analysis (Fig 3B). The production of NO by ZIKV-infected microglia did not change at 6, 12 and 24 hpi, but was significantly induced at 48 hpi (P<0.05) (Fig 3C).

### ZIKV infection modulates lysophospholipid and phospholipid production in microglial cells

**Fig 4:**
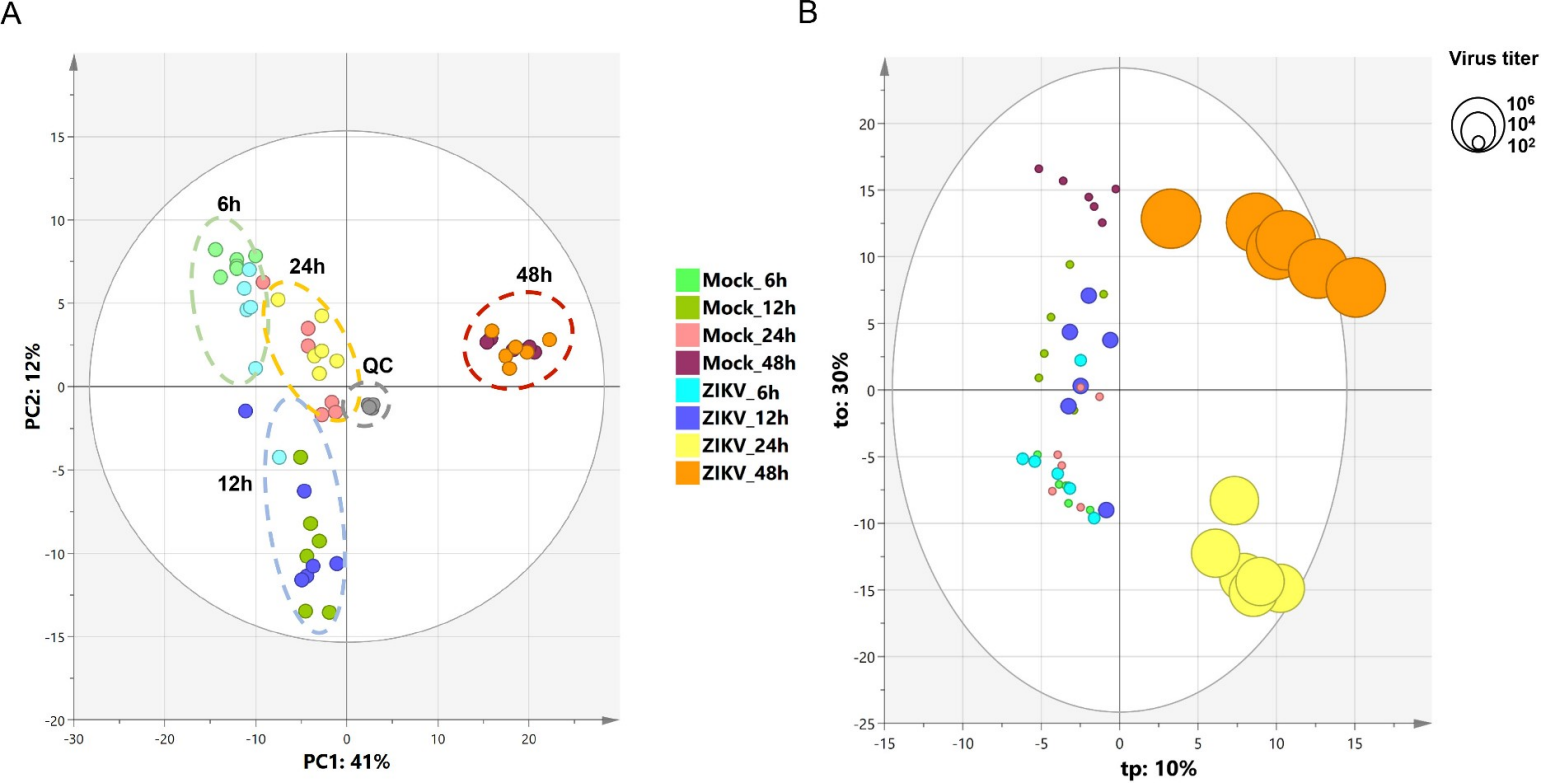
Principal component analysis and orthogonal projection to latent structure regression. (A) PCA score plot of the LC-HRMS dataset scaled to unit variance. (B) OPLS score plot of LC-HRMS dataset with virus titration level as Y input. Circle diameters are scaled according to virus titer.

**Fig 5:**
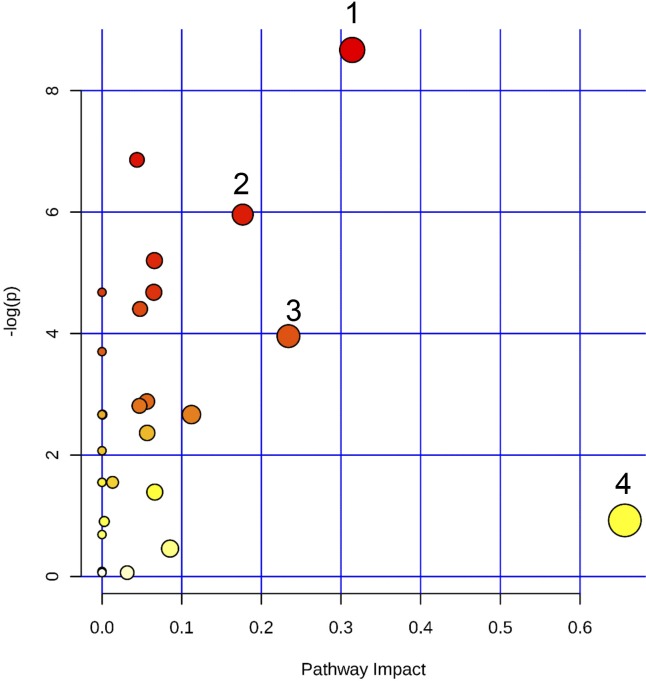
Pathway enrichment analysis using all identified features from the LCMS dataset. (1) Glycerophospholipid metabolism; (2) Alanine, aspartate and glutamate metabolism; (3) Arginine and proline metabolism; (4) Linoleic acid metabolism.

**Fig 6:**
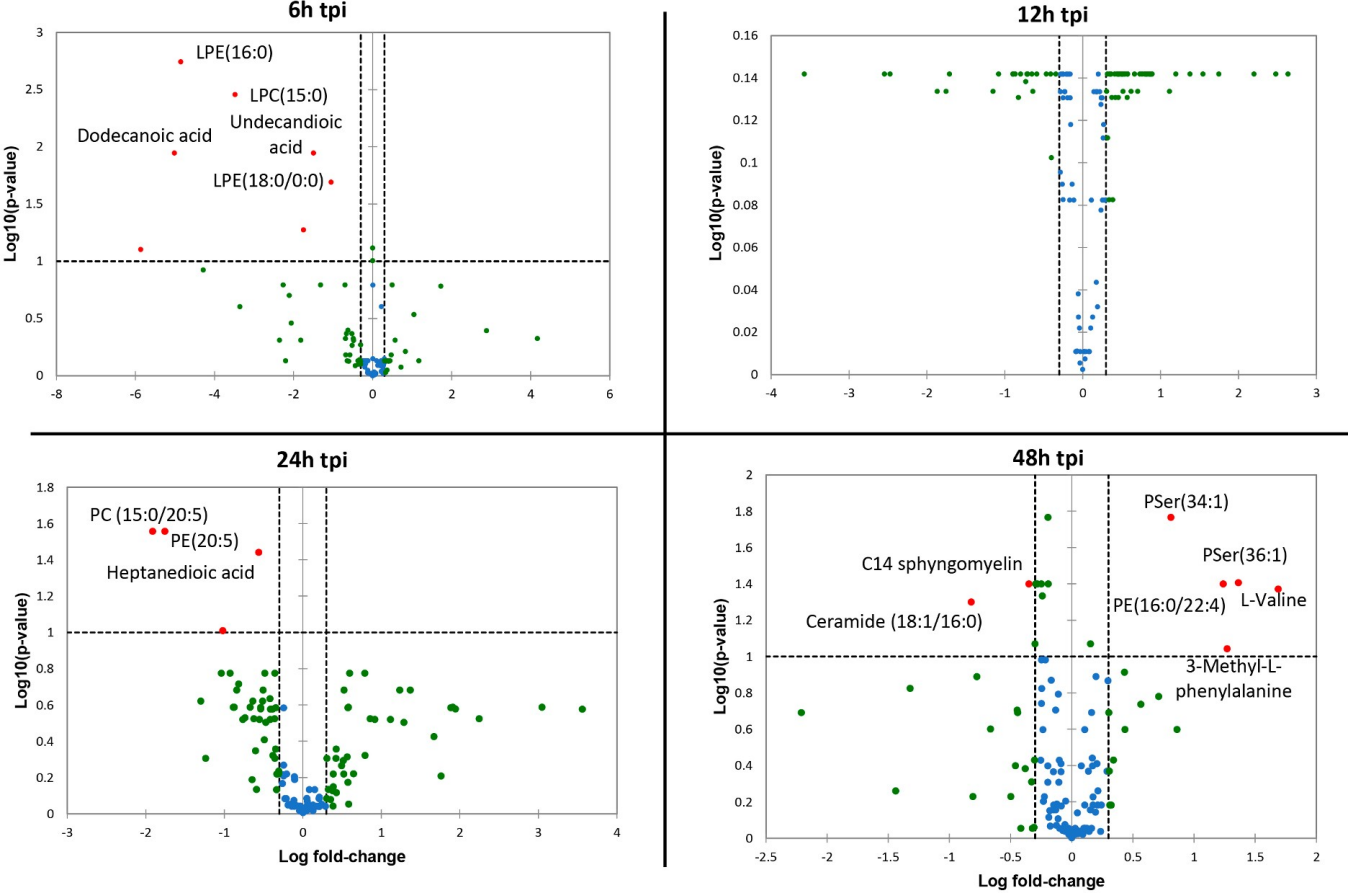
Volcano plot using all identified features with VIP score > 1. The volcano plot was depicted as a log scaled axes of fold change (x-axis) and p-value from impaired t-test (y-axis). Dashed line delineates metabolite with fold change >1.5 and *p*-value < 0.05. Metabolite IDs were displayed for marked up regulated (right side) or down regulated compounds (left side). tpi: time post infection.

**Fig 7:**
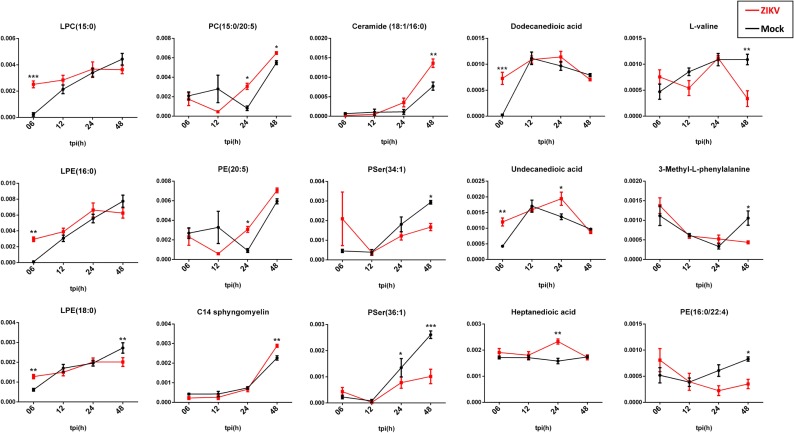
Significative variation of identified metabolites from 6 to 48 hpi expressed in relative mean peak area. Bars display standard error mean (SEM), stars indicate significative differences from tow way ANOVA and Sidak post hoc test (alpha = 0.05; * < 0.05; ** < 0.01; *** < 0.001). tpi: time post infection.

To identify the lipid metabolism associated with the regulation of the inflammatory process that results from ZIKV infection of human microglia, we performed a metabolomic analysis of microglial cells infected with the French Polynesian ZIKV strain. CHME-5 cells were infected with ZIKV at MOI 1, harvested at 6, 12, 24 and 48 hpi. To ensure sufficient statistical power, six independent replicates per conditions were performed and analyzed by Liquid Chromatography-High Resolution mass Spectrometry (LC-HRMS). The resulting data matrix accounting for 580 features (*mz*/rt pairs) and 48 samples, in addition to 5 QCs samples, was treated by multivariate data analysis. In order to obtain an unsupervised overview of the whole dataset, principal component analysis (PCA) was applied. The PCA score plot (Fig 4A) clustered each biological replicate together while QC samples centered on plot. This general pattern confirms the reproducibility of manipulations and robustness of data acquisition. The first principal component axis (PC1) which accounts for 41% of the total variance clearly separates each time point. The PC2 separates the kinetic points at 12 and 24 hours after initial infection. Mock and ZIKV-infected samples were not separated on this PCA score plot. Since most of the variance observed in the LC-MS dataset was ruled through kinetics of cell growth, a supervised model based on virus titer level as a discriminative variable (Y input) was applied. The OPLS regression model allowed features ranking according to their positive or negative correlation with virus infection level (Fig 4B). The quality of model prediction was satisfactory (R2Y = 0.8, Q2Y = 0.5, CV-Anova *p-value* = 0.003) and the permutation plot assessed its validity (S1A Fig). Then, variable influence on the projection (VIP) plot (S1B Fig) of the OPLS model was used to select features with a VIP score above 1 for further data analysis. In order to obtain an overview of most impacted pathways upon ZIKV infection, a pathway enrichment analysis was afforded using all identified features with a VIP score above 1 (Fig 5) using a regression model with titration level as Y value. Finally, to focus on most relevant metabolites, all selected features were filtered according to their respective ratio between mock and ZIKV-infected cells and impaired t-test *p-value* to build a kinetic volcano plot for each time point post infection (Fig 6). Overall, the data gathered using this metabolomic approach indicate that ZIKV infection modulates glycerophospholipid, amino acid and, to a lesser extent, linoleic acid pathways in microglial cells. Our kinetic study also revealed a peculiar dynamics of the microglial cell metabolome following ZIKV infection. From 6 to 24 hpi, the expression of several metabolites was upregulated (Fig 6, right side), including that of LPC, lysophophatidylethanolamines (LPE), phosphatidylethanolamine (PE), phosphatidylcholine (PC), Ceramide and Sphingomyelin. In contrast, at 48 hpi a significant decrease of phosphatidylserine (PSer) levels (Fig 6, left side) is observed. In particular, the results show that levels of LPC(18:2) and LPE(18:0), were significantly increased at early time points post-infection (Fig 7). In addition, levels of PC (15:0/20:5) and PE(20:5) were increased in ZIKV-infected cells at 24 hpi and 48 hpi (Fig 7). The levels of two sphingolipid, C14 sphingomyelin and ceramide (18:0/20:5), were increased at 48 hpi as well (Fig 7). ZIKV also upregulated the synthesis of carboxylic acids such as dodecanedioic and undecanedioicacid that were detected at elevated levels in infected cells, as compared to mock-infected cells, at 6 hpi, whereas heptanedioic acid was detected at 24 hpi (Fig 7). In contrast, levels of metabolites related to to PSer were decreased in ZIKV-infected Microglial cells, as compared to mock-infected cells. ZIKV infection resulted in a decreased synthesis of PSer(36:1) and PSer(34:1) detected at 48 hpi. Levels of PE(16:0/22:4) and the amino acids L-valine and 3 methyl-L-phenylalamine were also significantly decreased in ZIKV infected cells at 48 hpi (Fig 7).

## Discussion

The emergence of ZIKV has been associated with increased microcephaly in South America and French Polynesia. Although it has been reported that ZIKV is able to infect microglial cells and that infection results in increased centrosome numbers and impaired spindle positioning, thus potentially contributing to microcephaly [14], the metabolomic profile of ZIKV-infected glial cells remains to be determined. The results obtained in the present study indicate that ZIKV infection induces neuroinflammation in microglia which leads to the production of IL-6, TNF-α, IL-1β and IFNs, molecules with strong pro-inflammatory effects that feature among the most potent neuroinflammatory cytokines. This finding corroborates an earlier report in the literature that shows the expression of these cytokines in primary fetal brain tissue of ZIKV-infected donors [13]. These results also show that the CHME-5 cell line that has been derived from human fetal microglia [25] and used in the present study is a relevant physiological model to study the neuro-inflammation induced by ZIKV. They furthermore confirm and extend the observation that flavivirus infection induces the production of a wide array of pro-inflammatory mediators in microglial cells, as shown previously in an experimental animal model of Japanese Encephalitis Virus [32]. We also demonstrated in the present study that ZIKV increases the production of iNOS and NO in microglial cells during infection. It has been demonstrated that activated microglia are able to cause neuronal death via the production of high levels NO [33] and the production of NO in ZIKV-infected cells could therefore contribute to neurological disorders caused by ZIKV. In addition, our results show that ZIKV induces the expression of transcripts for CX3CR1, a chemokine receptor that is required for maintaining proper microglial functioning [34]. Signaling through CX3CR1, following interaction with its ligand CXCL1, determines microglial cell function during the development of the CNS [35]. However, the role of CX3CR1 and its ligand CX3CL1 during ZIKV infection and its effect on the interaction between neurons and microglia at the synapse have not been identified yet.

LC-HRMS analyses were conducted to study the association of various lipid metabolites with the infection of microglial cells with ZIKV. LPC was found to be up regulated by ZIKV in microglial cells. These fatty acid chains are involved in the alteration of membrane structures and can mediate acute inflammation throughout the vasculature and local tissue sites [36]. It has been demonstrated that both *in vitro* and *in vivo* exposure of microglial cells to LPC induces a transformation from a ramified into an ameboid morphology [37]. Because activation of microglial cells is accompanied by cell deramification, it has been suggested that LPC leads to the activation of microglia [38]. Indeed, it has been demonstrated that LPC stimulates processing and release of IL-1β from microglia [39], an observation that is supported by the findings of the present study. It can be hypothesized that ZIKV infection-induced production of LPC by microglia in its turn increases the production of IL-1β and stimulates cell proliferation thereby inducing neuroinflammation. This lysophospholipid is also involved in neuroinflammation by activating the inflammasome pathway in microglia [40].

Our study identifies LPE in cells infected with ZIKV already at 6 hpi. The physiological significance of cytoplasmic LPE in microglia remains unknown, whereas the role of LPE(18:0) in neuroinflammatory activity has not yet been documented either.

Our results showing that PC expression is induced in ZIKV-infected glial cells corroborates similar findings using a lipodomics approach in ZIKV-infected mosquito cells [41]. This lipid class has previously been associated with cell activation during infection with positive strand RNA viruses, resulting in an increase or accumulation of PC [42]. It is of note that PC is utilized in the formation of viral particles and synthesized at the site of the viral replication complexes [42]. Our study demonstrates that ZIKV upregulates PE(20:5) in infected-cells at 24 hpi. PE is an anionic lipid that is normally found in the inner side of plasma membranes [43] and that is synthesized *via* a decarboxylation reaction of PSer. Interestingly, it has been reported that virion-associated PE promotes TIM1-mediated infection by Ebola, Dengue, and West Nile viruses and that binding of the viral envelope by TIM receptors contributes greatly to flavivirus internalization [44]. In contrast however, the synthesis of PE(16:0/22:4) is dowregulated in microglia following ZIKV infection and it is therefore of interest to determine the role of this PE metabolite in virus mediated neuroinflammation.

The sphingolipids C14 Sphingomyelin and Ceramide determine the biophysical properties of cell membranes that play an important a role in different processes such as cell differentiation and regulation of apoptosis [45]. Both metabolites were detected during ZIKV infection and their presence could be due to active sphingolipid biosynthesis which is required for Flavivirus infection of cells [46]. It has been shown that ceramides mediate the production of inflammatory cytokines through phosphorylation of JNK, whereas Ceramide (16:0) activates Toll-Like Receptor 4 and the inflammasome, which results in the production of TNFα, IL-6, and IL-1β [47]. Therefore, we hypothesized that the induction of such proinflammatory cytokines in ZIKV-infected microglial cells is likely to be mediated by sphingolipids.

ZIKV infection also depletes cells of a subset of metabolites, including Pser, which is the major glycerophospholipid in the brain. As has been demonstrated for West Nile, Dengue and Ebola viruses [48], PSer is essential for viral replication, through binding of the virus to target cells or phagocytosis of viral particles. Furthermore, PSer is a substrate for TAM and CD300a receptors which are known to be specific for ZIKV-mediated internalization into the cell. Surprisingly, expression levels of both lipid metabolites, PSer(34:1) and PSer(36:1), decreased in infected cells at 48 hpi. We hypothesize that PSer is preferentially used or over-used by ZIKV for production and replication upon viral infection until depletion. Altenatively, it can be hypothesized that in order to combat infection, the synthesis of Pser is decreased in microglial cells that allows for an increase in the production of NO and TNF-α. This possibility is underscored by the finding that the production of both inflammatory mediators was profoundly inhibited when microglial cells were exposed to PSer-liposomes [49].

It has been shown that dicarboxylic acids are important substrates in the central nervous system [50]. Indeed, our results show that the synthesis of the two dicarboxylic acids Undecanedioic (C11) and Dodecanedioic (C12) acid was rapidely increased in ZIKV-infected cells, in contrast to Heptanedioic (C7) acid which was elevated at 24 hpi. Moreover, the expression of phenylalaninmethyl and valine were significantly decreased in cells at 48 hpi. Although the effect of depletion by ZIKV was observed, the function of these amino acids in flavivirus infection is not documented; further studies should be carry out to confirm if these metabolites participate in their own macromolecular synthesis.

Taken together, our finding describe an important role for microglia in neuroinflammation and lipid metabolism during ZIKV infection. The synthesis of lipid metabolites by these cells may contribute to the production of pro-inflammatory cytokines that play a crucial role in ZIKV-induced neuroinflammation.

## Supporting information

S1 Fig (A) Permutation plot of OPLS regression model; (B) VIP plot (Variable importance for projection) of the first 150 features.
(JPG)

S1 Table Processed raw data. Concatened and processed raw data using MSDial (sheet 1) with selected biomarkers (sheet 2) described in Figs 6 and 7.
(XLSX)
