# Supplementary figures and images for: Zika virus infection modulates the metabolomic profile of microglial cells
Source: PLoS One. 2018 Oct 25;13(10):e0206093. doi: 10.1371/journal.pone.0206093 (PMC6201926; doi:10.1371/journal.pone.0206093)

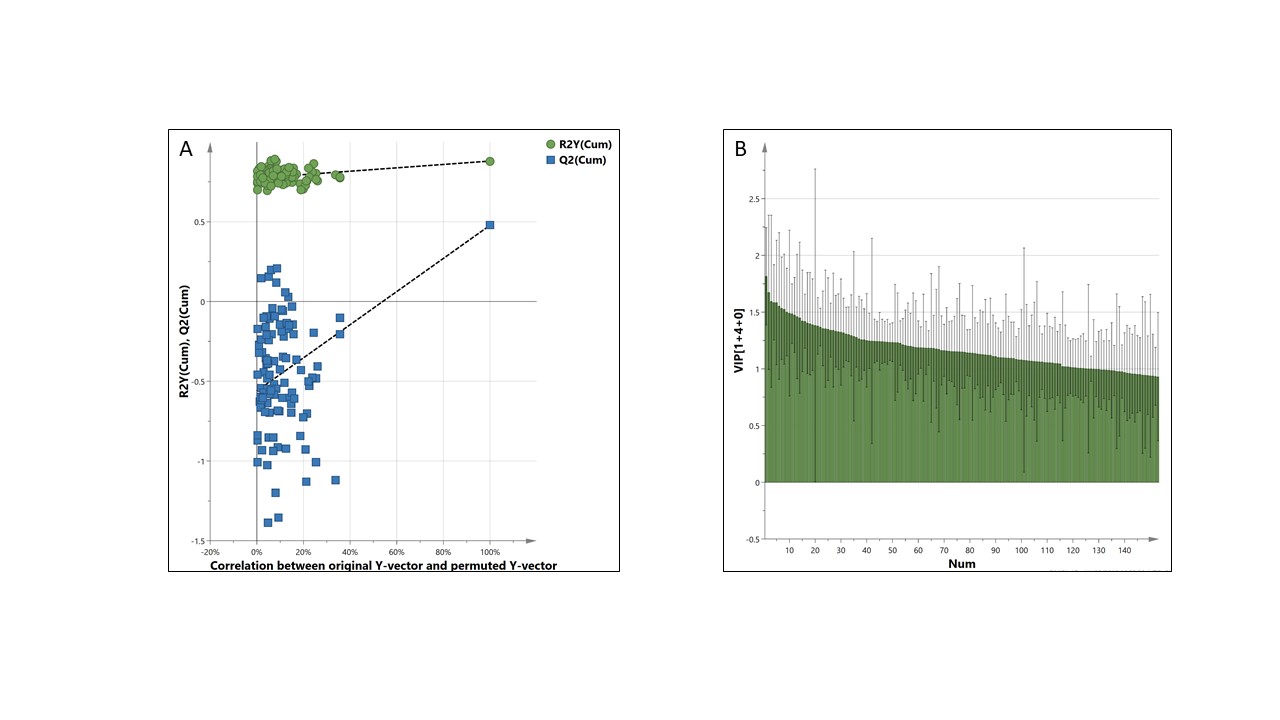

Supplement: S1 Fig — (A) Permutation plot of OPLS regression model; (B) VIP plot (Variable importance for projection) of the first 150 features. (JPG) [file pone.0206093.s001.jpg]
